# Grape Cultivar Features Differentiate the Grape Rhizosphere Microbiota

**DOI:** 10.3390/plants11091111

**Published:** 2022-04-20

**Authors:** Lijun Bao, Bo Sun, Yingxue Wei, Nan Xu, Shiwei Zhang, Likun Gu, Zhihui Bai

**Affiliations:** 1 Key Laboratory for Heavy Metal Pollution Control and Reutilization, School of Environment and Energy, Peking University Shenzhen Graduate School, Shenzhen 518055, China; baolijun@pku.edu.cn (L.B.); xunan@pkusz.edu.cn (N.X.); 2 Key Laboratory of Environmental Biotechnology, Research Center for Eco-Environment Sciences, Chinese Academy of Sciences, Beijing 100085, China; bosun_st@rcees.ac.cn (B.S.); w16619750024@163.com (Y.W.); zhbai@rcees.ac.cn (Z.B.); 3 College of Resources and Environment, University of Chinese Academy of Sciences, Beijing 100049, China; 4 College of Resources and Environment, Henan University of Engingeering, Zhengzhou 451191, China; 5 Xiongan Institute of Innovation, Xiongan New Area Baoding 071000, China

**Keywords:** grape variety, rhizosphere, microbiome, network analysis, high-throughput sequencing

## Abstract

Rhizosphere microflora are key determinants that contribute to plant growth and productivity, which are involved in improving the uptake of nutrients, regulation of plants’ metabolisms and activation of plants’ responses against both biotic and abiotic stresses. However, the structure and diversity of the grape rhizosphere microbiota remains poorly described. To gain a detailed understanding of the assembly of rhizosphere microbiota, we investigated the rhizosphere microbiota of nine grape varieties in northern China by high-throughput sequencing. We found that the richness and diversity of bacterial and fungal community networking in the root compartments were significantly influenced by the grape variety. The bacterial linear discriminant analysis showed that *Pseudomonas* and *Rhizobium*, which were considered as potential plant-growth-promoting bacteria, were more enriched in Pinot noir, and *Nitrosospira* was enriched in Gem. The fungal linear discriminant analysis showed that *Fusarium* was more enriched in Longan, *Sporormiella* was more enriched in Merlot, *Gibberella* and *Pseudallescheria* were more enriched in Gem and *Mortierella* was more abundant in Cabernet Sauvignon. The 16S rRNA functional prediction indicated that no significance differentiates among the grape varieties. Understanding the rhizosphere soil microbial diversity characteristics of different grape varieties could provide the basis for exploring microbial associations and maintaining the health of grapes.

## 1. Introduction

The rhizosphere is the soil compartment influenced by plant roots [1]. It is one of the most complex environments influenced by plant roots and microbes and is very important for plant functioning because it provides nutrients for plants and offers protection against pathogens, influencing plant health, development and productivity [2]. Mycorrhizal fungi, or nitrogen-fixing bacteria, play important roles in plant performance by improving mineral nutrition [3]. For instance, free-living nitrogen-fixing bacteria, such as *Azospirillum*, *Acetobacter diazotrophicus*, *Herbaspirillum seropedicae*, *Azoarcus* spp. and *Azotobacter*, and symbiotic nitrogen-fixing bacteria, such as *Rhizobium* and *Bradyrhizobium*, contribute to plants’ nitrogen availability and productivity [4,5]. The symbiotic relationship between the legume and rhizobium has been widely studied. The secretion of a cocktail of phenolic molecules in the legume rhizosphere, mainly flavonoids and isoflavonoids, which is taken up by rhizobia, bind the transcriptional regulator NodD and activate a suite of bacterial nodulation genes [6]. These nodulation genes (called Nod factors) are responsible for the production of lipochitooligosaccharides. Nod factors cause legume cell division and meristem formation, and rhizobia then infect plant roots through crack entry, intercellular colonization of epidermal cells or the formation of infection threads [7]. Rhizobia eventually enter the legume root cortical cells via endocytosis, where they differentiate into nitrogen-fixing bacteroids within a unique plant organelle called the symbiosome. The symbiosome is delimited by a plant-derived membrane that controls nutrient exchange between the symbionts [8]. Unlike roots, legume nodules have a peripheral vasculature [8,9]. Symbiotic and non-symbiotic plant-growth-promoting rhizosphere bacteria can also directly promote plant growth by producing plant hormones such as auxins, cytokinins, gibberellins, ethylene and abscisic acid [10]. Rhizosphere microbes can inhibit plant pathogens by producing fungal cell wall degrading enzymes, such as chitinase and beta-1, 3-glucanase [10,11]. Host plants can adjust environmental factors such as pH and soil nutrients to shape their rhizosphere microbiome [12]. Additionally, plant genotype may also greatly influence the structure of rhizosphere microbial communities [13,14]. In many cases, diseases are caused by the microbial community, while other microbes may antagonize phytopathogens, supply nutrients for different host plants and modulate plant growth [15]. Rhizosphere microbes were characterized based on the extraction of community DNA to illustrate the relationship between plants and their microbes. It has been reported that the roots of maize, wheat and rape carry various microbes as a consequence of the assimilation of root exudates [16].

The grapevine rhizosphere contains a large community of microorganisms that interact with plant organs; these microorganisms may be delivered to the winery and affect wine quality [17]. Recently, molecular biology studies have suggested that the grape microbiome is linked with the vineyard’s location, climatic and other environmental factors [2]. Using a traditional cultivable method as well as T-RFLP, Martins et al. expounded the epiphytic bacterial communities on the grapes, leaves, bark and soil of Merlot [18]. Applying sequencing analysis, Zarraonaindia et al. [19] showed that the Suffolk County Merlot soil microbiome influences the grapevine-associated microbiota.

Microbiological studies in the soil environment are performed at a small scale because the majority of soil bacteria cannot yet be cultured [2]. The Shacheng district is located in northern China and grows most varieties of wine grapes. Its cinnamon soil with a sandy texture and its hilly and mountainous areas make it very suitable for the growth of grapes. However, little is known about the microbial communities in the rhizosphere of the varieties of wine grapes in this district, which grows the most wine grape varieties in China. In this study, we used high-throughput sequencing to illustrate the microbial communities in northern China. The distribution of the microflora was investigated, and the results showed divergence among the varieties of grapevine.

## 2. Results

### 2.1. Richness and Diversity of Microbiome Communities Associated with the Root Systems of Different Grape Varieties

**Figure 1:**
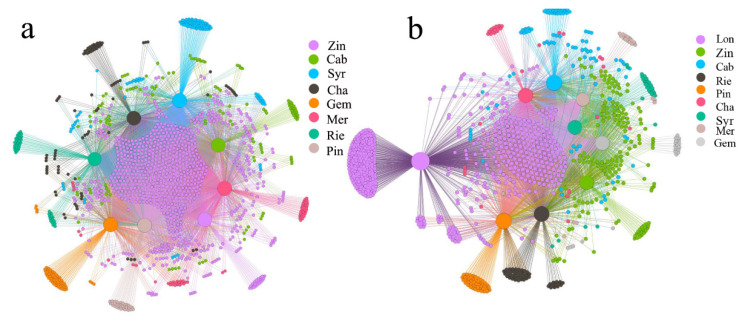
Bipartite network analysis of grape rhizosphere bacterial (**a**) and fungal (**b**) communities. Zinfandel (Zin), Cabernet Sauvignon (Cab), Syrah (Syr), Merlot (Mer), Gem (Gem), Pinot noir (Pin), Riesling (Rie), Longan (Lon), Chardonnay (Cha).

The rarefaction curves decreased slowly, indicating that the sequences were of suitable quality and could be further analyzed (Figure S1). On average, for bacterial communities in the grape rhizosphere samples, the sequences ranged from 52,256 to 85,644, and 3117 OTUs were obtained from the different varieties (Table S1). A bipartite network consisting of 1384 nodes and 6973 edges was generated for the rhizosphere of the nine different varieties of wine grape, and the modularity index was 0.073 (Table S2). In this network, Zin had the highest number of OTUs, followed by Cab, and Pin had the lowest (Figure 1a). For fungal communities, the sequences ranged from 222,674 to 282,519, and 320 OTUs were obtained from the rhizosphere. A bipartite network consisting of 1020 nodes and 4038 links was generated for the rhizosphere of the nine different varieties of wine grape, and the modularity index was 0.151(Table S2). In the fungal network, Lon had the highest number of OTUs, followed by Cab, and Mer had the lowest number (Figure 1b). The results showed that both bacteria and fungi have complex microbial community structures.

The coverage indexes for the bacterial communities and fungal communities were higher than 95% (Table 1) and 99% (Table 2), respectively. For the bacterial communities, there were no significant differences in the Simpson and Shannon indexes among the nine grape variety samples (Table 1). The ACE and Chao1 index of Mer and Pin were significantly different, while the four indexes of fungal communities were extremely significantly different (*p* < 0.05) among the nine wine grapes. Zin showed the highest alpha-diversity of the fungal communities (Table 2).

### 2.2. Grape Microbiome Distribution in the Rhizosphere of Different Varieties of Wine Grape

**Figure 2:**
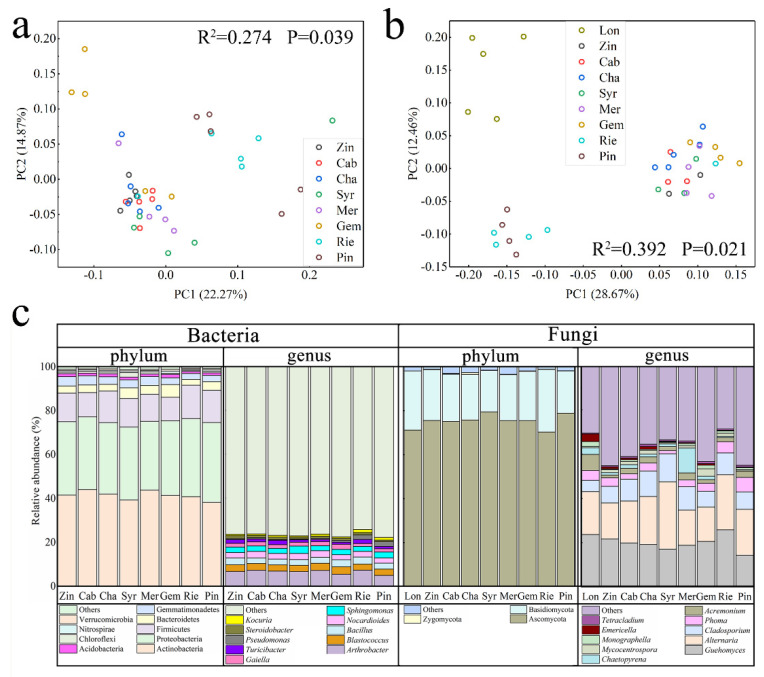
Principal coordinates analysis (PCoA) of the variation in bacteria (**a**) and fungi (**b**) community structures of nine types of wine grape rhizosphere. Relative abundance of bacteria and fungi associated with phylum and genus (**c**). Zinfandel (Zin), Cabernet Sauvignon (Cab), Syrah (Syr), Merlot (Mer), Gem (Gem), Pinot noir (Pin), Riesling (Rie), Longan (Lon), Chardonnay (Cha).

To compare the differences in total bacteria and fungi among the different varieties of grape rhizosphere, pairwise dissimilarities within the communities were visualized by PCoA. Dimensional scaling of the Bray–Curtis dissimilarity matrix to two dimensions revealed separation of the communities among the varieties in different samples, including the bacterial communities and fungi communities. PerMANOVA analysis also revealed that the varieties were weakly but significantly linked to rhizosphere bacterial and fungi community composition, respectively (*p* < 0.05) (Figure 2). Figure 2a showed that Mer and Pin grouped together, while Zin, Cab, Cha, Syr and Rie grouped together, Gem separately with other varieties. This showed that Mer and Pin was closer to each other in their bacterial community compositions, while Zin, Cab, Cha, Syr and Rie might have relatively similar community compositions. From the Figure 2b, Pin and Rie were closer to each other, Lon was separate from others, while Zin, Cab, Cha, Syr and Mer have similar fungi composition.

Comparing the bacterial communities among the rhizopsphere samples from different grape varieties showed that the abundances of the major phyla and genera were slightly different (Figure 2c). The nine dominant bacterial phyla in the rhizospheres of Mer, Syr, Zin, Cha, Gem, Pin, Rie, Lon and Cab were Actinobacteria, Firmicutes, Proteobacteria, Bacteroidetes, Gemmatimonadetes, Acidobacteria, Chloroflexi, Nitrospirae and Verrucomicrobia. Among them, the most abundant phylum was Actinobacteria, with the highest abundance in Cab (44.24%) and the lowest abundance in Pin (38.25%). The top ten dominant bacterial genera in the grape rhizosphere were *Arthrobacter*, *Blastococcus*, *Bacillus*, *Nocardioides*, *Sphingomonas*, *Gaiella*, *Turicibacter*, *Pseudomonas*, *Streptomyces* and *Kocuria*. Among them, *Arthrobacter* was the most abundant bacterial genus, with the highest abundance in Rie (7.19%), Cab (7.11%) and Mer (6.53%).

Comparing the fungal communities in the rhizospheres of different varieties of grape showed that many of the phyla and genera had varying abundances (Figure 2c). The three dominant fungal phyla in these varieties were Ascomycota, Basidiomycota and Zygomycota, which composed approximately 99% of the total phyla. Among them, the most abundant fungal phylum was Ascomycota in Syr (79.48%), and the lowest abundance of Ascomycota in Rie was also as high as 70.30%. The ten dominant fungal genera in these grape varieties were *Guehomyces*, *Alternaria*, *Cladosporium*, *Phoma*, *Acremonium*, *Chaetopyrena*, *Mycocentrospora*, *Monographella*, *Emericella* and *Tetracladium*, which composed approximately 50% of the total sequences. Among them, the most abundant fungal genus was *Guehomyces* in Lon (23.66%), and the lowest abundance of Guehomyces was 14.28% in Pin.

**Figure 3:**
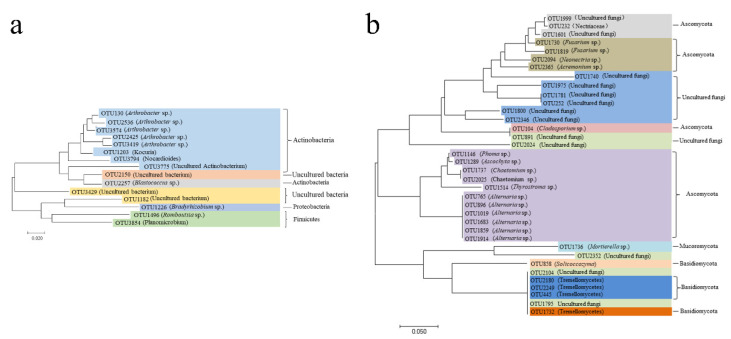
Phylogenetic tree of the core bacterial communities in the rhizospheres of nine grape cultivars (**a**). Phylogenetic tree of the core fungal communities in the rhizospheres of nine cultivars of grapes (**b**).

Eighteen bacterial core OTUs were obtained and found to constitute more than 60% of the total sequences (Table S3). When these OTUs were blasted against the NCBI database, the relative abundance of *Blastococcus* sp. (OTU2257) was found to be higher than others. The relative abundance of *Blastococcus* sp. was higher than 27.41%. *Arthrobacter* sp. contained the most OTUs (OTU130, OTU2425, OTU2536, OTU3419, OTU3574), and the relative abundance of *Arthrobacter* sp. was between 7.71% and 18.20%. The phylogenetic tree of the core bacterial communities showed that these genera were included in the bacterial phyla. The amount of each taxonomic group differed among the samples. Actinobacteria accounted for 13.75%, 14.68%, 14.30%, 12.92%, 15.10%, 11.84%, 14.68% and 12.78% in Zin, Cab, Cha, Syr, Mer, Gem, Rie and Pin, respectively. Firmicutes accounted for 1.85%, 1.45%, 2.24%, 2.17%, 2.16%, 1.08%, 2.15% and 3.55% in Zin, Cab, Cha, Syr, Mer, Gem, Rie and Pin, respectively. Proteobacteria accounted for 0.74%, 0.62%, 0.68%, 0.52%, 0.60%, 0.77%, 0.62% and 0.63% in Zin, Cab, Cha, Syr, Mer, Gem, Rie and Pin, respectively (Figure 3a). The results obtained above showed that most of the bacterial core of the OTUs constituting the grape rhizospheres were similar, and that most of the OTUs were *Arthrobacter* sp.

Thirty-six fungal core OTUs were obtained and found to constitute more than 60% of the total sequences (Table S4). When these OTUs were blasted against the NCBI database, it was found that among all the fungal core OTUs, the relative abundance of uncultured fungi was higher than those of the other OTUs. The relative abundance of uncultured fungi (OTU1795) was higher than 54.78%. *Alternaria* sp. contained the most OTUs (OTU765, OTU896, OTU1019, OTU1683, OTU1859), and the relative abundance of *Alternaria* sp. was between 5.63% and 18.20%. The phylogenetic tree of the core fungal communities showed that the fungal genera were included in three fungal phyla. The amount of each taxonomic group differed among the samples. Ascomycota accounted for 25.62%, 29.31%, 41.20%, 36.12%, 51.63%, 31.77%, 26.41%, 42.34% and 46.04% in Lon, Zin, Cab, Cha, Syr, Mer, Gem, Rie and Pin, respectively. Basidiomycota accounted for 6.44%, 12.62%, 4.25%, 9.88%, 6.91%, 7.17%, 5.14%, 15.96% and 4.06% in Lon, Zin, Cab, Cha, Syr, Mer, Gem, Rie and Pin, respectively. Mucoromycota accounted for 0.03%, 3.99%, 0.41%, 0.71%, 0.29%, 2.03%, 0.56%, 0.82% and 0.38% in Lon, Zin, Cab, Cha, Syr, Mer, Gem, Rie and Pin, respectively (Figure 3b). From the fungi core OTUs analysis, it was found that most of the grape rhizosphere also have similar constitutions, with an abundant genera of *Alternaria* sp. and *Fusarium* sp.

### 2.3. Differences in Bacterial and Fungal Diversity among Varieties

**Figure 4:**
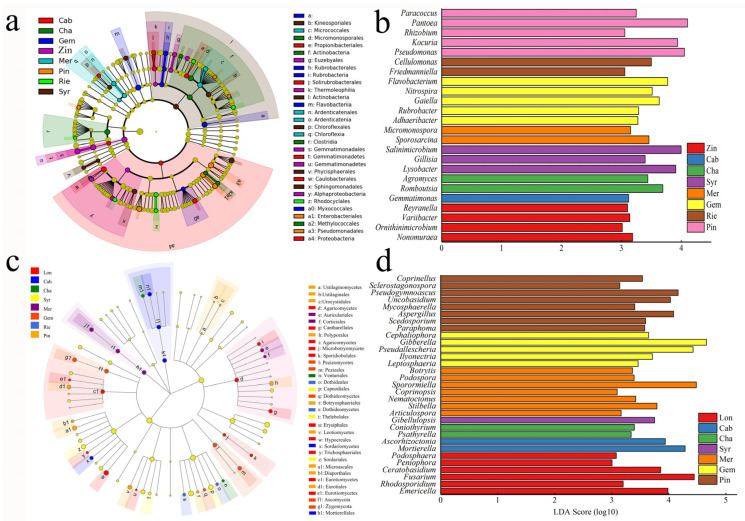
Cladograms suggesting the polygenetic distribution of bacteria (**a**,**b**) and fungi (**c**,**d**) in the rhizosphere of nine grape varieties as determined by linear discriminant analysis (LDA) effect size (LEfSe). Zinfandel (Zin), Cabernet Sauvignon (Cab), Syrah( Syr), Merlot (Mer), Gem (Gem), Pinot noir (Pin), Riesling (Rie), Longan (Lon), Chardonnay (Cha).

We used LEfSe to identify discriminative bacteria taxon among the different grape varieties. The LEfSe results for all species showed 30 bacterial taxa with significant differences. Cab and Syr were distinguished from others at the phylum level, with the significance enriched in Proteobacteria and Actinobacteria, respectively. Pin, Cha, Gem, Mer were distinguished from each other at the order level, with the significance enriched in *Enterobacteriales*, *Micromonsporales*, *Rubrobacterales*, *Micrococcales* or *Ardenticatenales* (Figure 4a). After analyzing differences among the varieties from the phylum to the genus, a closer look was taken into the differences in bacterial community structures at the genus level occurring over the different varieties. The rhizosphere bacterial taxa with the greatest differences over the eight wine grape varieties are displayed in Figure 4b. It was showed that Zin, Cab, Cha, Syr, Mer, Gem, Rie and Pin could all be clearly distinguished at the genus level, respectively. The number of enriched genera in Pin and Gem were higher than in Cab, Syr, Rie, Mer, Cha and Zin, while Lon nearly had no significantly enriched genus. In addition, *Pseudomonas* and *Rhizobium*, which are considered as potential plant-growth-promoting bacteria, were more enriched in Pin, and the potential ammonia-oxidizing bacterium *Nitrosospira* was enriched in Gem.

Similarly, we used LEfSe to identify distinctive fungi taxa among the different grape varieties. The LEfSe results for all species showed 34 fungal taxa with significant differences. At the phylum level, Zygomycota was significantly enriched in Cha, but its composition was distinguished at the order level (Figure 4c). In order to analyze the differences among varieties from the phylum to the genus, a closer look was taken into the differences in the fungal community structure at the genus level occurring over the different varieties. The rhizosphere fungal taxa with the greatest differences over the nine wine grape varieties are displayed in Figure 4d. It shows that Lon, Cab, Cha, Syr, Mer, Gem and Pin could all be clearly distinguished at the genus level, respectively. The number of enriched genera in Pin was higher than in the other six grape varieties, followed by Mer, while Rie and Zin nearly had no significantly enriched genera. In addition, *Fusarium* was more enriched in Lon, *Sporormiella* was more enriched in Mer, *Gibberella* and *Pseudallescheria* were more enriched in Gem, and *Mortierella*, which can produce polyunsaturated fatty acids, was more enriched in Cab [20].

To elucidate the differences in the bacterial and fungal composition among the different grape varieties, Bray–Curtis and unweighted Unifrac dissimilarity were adopted to dissect the varieties’ relationships. We calculated the taxonomic metric (calculated from OTUs), and tests showed that community structure varied among the different varieties, exerting a significant impact on bacterial Bray–Curtis (R_adonis_ = 0.319, *p* < 0.01; adonis, permutational multivariate analysis of variance; R_anosim_ = 0.317, *p* < 0.01; aonsim, analysis of similarities) and unweighted Unifrac (R_adonis_ = 0.215, *p* < 0.01, R_anosim_ = 0.245, *p* < 0.01) dissimilarity (Table 3).

For the fungal composition, tests showed that community structure varied among the different varieties and showed a significant impact on fungal Bra–Curtis (R_adonis_ = 0.373, *p* < 0.01, R_anosim_ = 0.295, *p* < 0.01) and unweighted Unifrac (R_adonis_ = 0.442, *p* < 0.01, R_anosim_ = 0.401, *p* < 0.01) dissimilarity (Table 4). These results revealed that the grape variety plays a significant role in shaping the bacterial and fungal communities, and varieties can be identified by the proportions of several key bacterial and fungal taxa.

### 2.4. Functional Genomics of Grapevine Rhizosphere Community

A functional characterization of amplicons was performed to learn about the physiological capabilities of the microbial communities and to link taxonomic shifts with functions. The differential abundances of KEGG orthologs (KOs) identified the key genotypic features of the grapevine rhizosphere microorganisms. In total, 193 different KOs were detected and organized into 39 small metabolic pathways at KEGG level 2 and 239 metabolic subsystems at KEGG level 3. These metabolic pathways were included in six basic metabolic systems at KEGG level 1: metabolism, genetic information processing, environmental information processing, human diseases, cellular processes and organismal systems. The main metabolic pathways were considered those with OTU sequences representing more than 5% of all the sequences in the grape rhizosphere samples for all the varieties. As shown in Table S8, 24 main metabolic pathways at level 2 were found. The top three metabolic pathways were membrane transport, amino acid metabolism and carbohydrate metabolism, with total relative abundances of 97.79%, 91.11% and 86.79%, respectively. A total of 53 main metabolic pathways at level 3 were found, and the highest 3 were transporters, ABC transporters and general function prediction only, with total relative abundances of 49.96%, 30.68% and 27.43%, respectively (Table S9). Microbial enzyme catalysis determines the functions of rhizosphere communities. Therefore, most KOs were related to the enzyme commission number. The most common of 32 enzymes found were drawn in a heatmap (standardized and normalized by Z-score) to further visualize the functional variation in the rhizosphere microbial communities (Figure S2). From the heatmap, we can see that enoyl-CoA hydratase (4.2.17), error-prone DNA polymerase (2.7.7.7) and aldehyde dehydrogenase (NAD+) (2.3.19) were the top three enzymes, and were more enriched in all of the varieties.

## 3. Discussion

Microflorae exist in complicated associations with crops and have key roles in shaping soil quality and improving crop health as well as its productivity. The origin of the microbes involved in wine fermentation is still poorly understood; they are commonly assumed to come from the grapes themselves. Many bacteria and fungi actively colonize the rhizosphere and have metabolic activities that modulate plant health or suppress disease-causing pathogens. Previous studies have attempted to determine the factors that significantly influence the rhizosphere microbiome. Some of these studies suggest that geographical location influences the community composition [21,22,23], some studies suggest that agricultural management practices play key roles in the community structure [24,25], accompanied by studies that exposed that seasonal changes are strongly correlated with shifts in the microsystems [26], while other studies suggest that the host plant’s species is the primary factor driving the community [21,27]. Despite the host plant improving our knowledge, more species observed in the rhizosphere are still unexplained. Our study covered nine varieties of wine grapes in north China to identify the variety factors that potentially play roles in the population, diversity and taxonomic composition. The results indicated that the composition of community structures was influenced by the variety, whereas some of the varieties did not appear to have features that differentiated them in the community.

Grape varieties were a strongly correlating factor to populations, diversity and the taxonomic composition of the microbiome community, including the bacterial and fungal community. Bokulish et al. [21] found that Chardonnay, Zinfandel and Cabernet exert some effect on community structure across the regions and vintages. Zarraonaindia et al. [19] reported that the structure of soil and other parts of the grapevine indicated the importance of soil for plant-organ-associated bacterial taxa. In our study, the OTU distribution showed the bacterial and fungal structures’ respective clustering, and combined together, indicate that they all have same origin and no differences in structure, but do in their populations (Figure 1). The alpha-diversity (OTU diversity, richness and evenness) of the nine varieties was weak or not significantly differentiate from each other. These microorganisms identified on the varieties come from the same vineyard, and most of the grapes were also planted in 1979, while cleft grafting on the same rootstock and a long-term equal environment make less difference in diversity among the varieties. These bacteria and fungi may migrate from the surrounding soil and airborne environment [19,28,29]. Bokulish et al. [21] stressed the importance of *terroir* through comparing regional microbial biodiversity in *Napa* and *Sonoma*, though our study chose only one place, which can also be considered as a part of *terroir*; the bacterial and fungal communities have no diversity differentiate, which may prove the significant role in the *terroir* indirectly. As for the composition of communities, the rhizosphere samples from varieties showed that the major phyla and genera were similar though had different abundances (Figure 2). We found that most varieties of grape rhizospheres were similar in their composition of bacterial core OTUs, and that most of the OTUs were *Arthrobacter* sp. and *Blastococcus* sp. *Arthrobacter* sp. are abundant in the soil, with high genetic adaptability [30,31], and have the potential for bioremediation [32,33]. Members of the genus *Blastococcus* distribute in various environments, and are considered as pioneers in extreme environments, such as rocks and desert sandy soils [34,35]. Perazzolli et al. [36] found that *Acetobacter* existed on all grapevine plants. *Alternaria* produces alternariol, alternariol monomethyl, altenuene, altertoxin and tenuazonic acid; these metabolites exhibit some degree of toxicity to mammalian and bacterial cells as well as to higher plants [37]. *Fusarium* sp. are involved in wine making and can produce pectinase, raising juice yields during the process of wine making [38,39]. In our study, these two potential functional microorganisms existed in nine varieties of wine grapes.

However, several differences were found in terms of beta-diversity, population and interactions within rhizosphere bacteria and fungi among the nine varieties. Due to variety differences, the PCoA and PerMANOVA analysis showed the variety change was the most important factor affecting the change in the community structure for the total bacterial and fungal communities. As for the bacterial and fungal communities’ population and composition, some phyla and genera showed significant differences. At the phylum level, Actinobacteria was significantly enriched in Syr compared with other eight varieties, while Proteobacteria was significantly enriched in Cab, which was in accordance with previous studies on Cab [21]. At the genus level, the number of enriched genera in Pin and Gem were higher than in Cab, Syr, Rie, Mer, Cha and Zin, while Lon nearly had no significant genus. Lon is a unique grape in China, which can be considered as both a wine grape and a table grape, and it may be the features of brewing and table that led to it not being extinguished from other varieties. *Pseudomonas* and *Rhizobium* were more enriched in Pin. Studies also found that *Pseudonmonas* can grow under extreme conditions [40,41]. Microflorae are closely interconnected to plants; for example, plants transfer carbon to bacteria and fungi, while microflorae improve phosphate and nitrogen accessibility as well as perform other nutrient acquisition tasks for the plants [42,43], and other studies also investigated the relationships among soils and other parts of the grapevine [18,44]. Grape cultivars differ in growth habit, and this invasion might explain how Vitis manages its grape-surface susceptibility to disease pressures in different environments [45]. The late-maturing characteristics increase the survival probability of *Pseudonmonas*, and could also explain why *Pseudonmonas* is enriched in Pin.

Zhang et al. [44] found that the microflora on leaves and grapes mainly originated from the soil, and these microflora ultimately form part of the juice and participate in fermentation. Zarraonaindia et al. [19] found that the Mer soil and root were dominated by *Proteobacteria* spp., *Acidobacteria* spp., *Bacteroidetes* spp., and *Verrucomicrobia* spp., and the reason for this difference may be the climate. Bokulich et al. [21] reported the importance between wine grapes and climate. In our study, though not comparing the influence of climate, such as seasons and growth periods, all the varieties were collected in the same time and same growth period, while controlling other factors indirectly. Marzano et al. [46] observed *Amnibacterium*, *Methylobacterium*, *Hymenobacter*, *Sphingomonas* and *Thermomonas* in Cab, which is consistent with the theory mentioned above; most of these genera come from the soil, and therefore, we can infer that the Cab microflora in that case was significantly richer in *Amnibacterium*, *Methylobacterium*, *Hymenobacter*, *Sphingomonas* and *Thermomonas*. In our study, *Propionibacteriales*, *Solirubrobacterales*, *Gemmatimonadales*, *Gemmatimonadetes*, *Caulobacterales* and *Proteobacteria* were significantly enriched in Cab. These results also indirectly proved the key role of environmental conditions. In addition, we compared the differences among the varieties using statistical methods and found that the grape variety significantly influenced the taxa in the grape microbial community. These results were consistent with the previous study [47].

Root exudate compositions are considered to explain the plant-specific microbial communities associated with the rhizosphere among the varieties. Functional redundancy is crucial for maintaining the balance of a functioning ecosystem [48]. Marasco et al. [25] showed that cultivars grafted onto the rootstocks represent the soil and that root endosphere partially have no potential function. In our study, PICRUSt functional predictions showed there were some similarities in the utilization of root secretions, and no significance function differentiate. How the microorganisms in different varieties influence the root function is deserving of further research.

## 4. Materials and Methods

### 4.1. Site Description and Sample Collection

This study was performed in a grape-growing region in Huailai County, and the geographic coordinates of the sampling sites recorded by a handheld GPS device (Magellan, Santa Clara, CA, USA) were 40°4′–40°35′ N, 115°16′–115°58′ E. The winery was built in 1979, and most of the grapes were also planted in 1979, and the average tree age is 28 years. The vineyard contains as many as 16 cultivars, including Zinfandel (Zin), Cabernet Sauvignon (Cab), Syrah( Syr), Merlot (Mer), Gem (Gem), Pinot noir (Pin), Riesling (Rie), Longan (Lon), Chardonnay (Cha), and also has other varieties, such as Midknight Beauty, Traminer, Chenin blanc. The grapevine region is about 75 hectare, most of the grapes were Rie, which can brew dry white wine, and other varieties accounted for almost the same proportion except Midknight Beauty, Traminer, Chenin blanc. All of the varieties were growing in the same soil type. No bactericide or insecticide was applied in the vineyard, and no chemical fertilizer was applied (Tables S5 and S6). All the selected grapes were cultivated in the same vineyard field, which was characterized by a clay-rich soil. We chose the widespread nine varieties of Zin, Cab, Syr, Mer, Gem, Pin, Rie, Lon as well as Cha, which abounds in the vineyard, with the rootstock of all the varieties being the same and differing in many characteristics, including their bunch and berry traits. In order to minimize cross contamination, such as by wind, rain wash, and roadside traffic activities, all the samples were taken in the middle of the corresponding vineyard. Samples of the nine varieties of grapes were collected at the veraison, which corresponds to stage 35 in the modified E-L system for identifying major and intermediate grapevine growth stages. At the time of sampling, the outside temperature was as follows: the maximum temperature was 29 °C, and the minimum temperature was 16 °C, the entire day was cloudy, the wind power was smaller than level three, and no rain came in the week before sampling (Table S7). Fresh soil was collected at about 10–15 cm depth close to the stem as soon as possible, but not in a way that damaged the stem. The fresh soil was passed through a 1-mm sieve to remove plant residues and stones [19,49]. Considering the heterogeneity of the tested rhizosphere, the soil samples were collected from at least five plants to form a composite sample, and five composite samples of rhizosphere were collected for each variety of grape. In total, 45 grape samples were collected.

### 4.2. DNA Extraction and Sequencing

For each soil sample, 0.5 g was weighed for DNA extraction. Genomic DNA was extracted from all the samples above using the FastDNA SPIN Kit for Soil (MP, Santa Ana, CA, USA), as described by the manufacturer’s handbook [2]. The bacterial 16S *rRNA* gene V5–V7 region primers 799F (5′-AAC MGG ATT AGA TAC CCK G-3′) [50] as well as 1193R (5′-ACG TCA TCC CCA CCT TCC-3′) were used, and the fungal internal transcribed spacer ITS1 was amplified with the primers (5′-CTT GGT CAT TTA GAG GAA GTAA-3′) and (5′-GCT GCG TTC TTC ATC GATGC-3′) [51]. The reverse primer was modified to contain a barcode [52]. PCR contained 5 μL 10 × Pyrobest Buffer (Takara, Maebashi, Japan), 1 μL DNA template, 2 μL of each primer (10 μmol/L), 4 μL dNTPs (2.5 μmol/L), 0.3 μL Pyrobest DNA Polymerase (2.5 U/μL, Takara, Japan), and water up to 50 μL. The PCR procedure was 94 °C for 3 min, 94 °C for 45 s, 50 °C for 60 s and 72 °C for 90 s for 35 cycles, and a final extension of 72 °C for 10 min. The reaction products were purified using an Ultra Clean PCR clean-up kit (Mobio, Solon, OH, USA). Then, the the purified reaction products were divided into equal two parts, one for bacterial sequencing and the other for fungal sequencing, for a total of 90 sequencing samples. After the amplicons were subjected to library preparation, the products were sequenced using an Illumina HiSeq PE250 paired-end (HiSeq 2500, PE250). One of the bacteria samples (Lon) was not sequenced successfully. The sequencing data sets of the bacteria and fungi were deposited in the NCBI’s Sequence Read Archive with the Accession Numbers SRP216299 and SRP216297, respectively.

### 4.3. Data and Statistical Analysis

The software package Mothur (version 1.36.1) was applied for sequence analysis following the standard operating procedure outlined at http://www.mothur.org/wiki/Schloss_SOP, accessed on 21 October 2019 [53]. All raw fastq files were quality-filtered using Trimmomatic [54] and assigned to their respective samples according to their unique nucleotide barcodes. Chimeric sequences were identified and removed using UCHIME. After the removal of barcodes and primers, pair-ended sequences were merged using FLASH [55]. The sequences were clustered into operational taxonomic units (OTUs) with a sequence threshold of 97% similarity, and putative chimeric sequences were removed using UPARSE algorithms in Usearch 7.0 at the 0.03 level [56], while representative sequences of OTUs were picked up simultaneously. The singletons and chimeras were filtered during the UPARSE procedure. For fungi, any sequence classified as nonfungal Eukarya was removed from further analysis.

The Majorbio cloud platform was applied to define the OTU rarefaction curves. Species richness and diversity indices (ACE, Chao1, Shannon and Simpson) were also calculated by the majorbio. Community similarities were based on OTU using a principal coordinates analysis (PCoA) based on Bray–Curtis distance matrices as well as PerMANOVA analysis. A linear discriminant analysis effect size (LEfSe) was applied to the OTU table (non-parametric factorial Kruskal–Wallis (KW) sum-rank test *p* < 0.05, LDA > 3.0; http://huttenhower.sph.harvard.edu/galaxy/, accessed on 15 November 2019, LDA, linear discriminant analysis) to identify the discriminant bacterial and fungi clade [57]. A co-occurrence network analysis was performed for each microbiome associated with the rhizosphere to expound the significant relations among the OTUs [58]. To build the network, we filtered out the OTUs with frequencies less than 0.05 and with spearman correlation coefficients higher than 0.85 [59], and visualized it using Gephi 0.9.2. The OTUs accounted for more than 5% of the total sequences considered as core OTUs. The phylogenetic tree of the core OTUs was generated with Mega 6. Using the 16S rRNA gene data, the function of the rhizosphere communities was predicted by PICRUSt [60]. The KEGG database was selected and used to predict molecular functions, and the relatives of enzymes larger than 3.0% were drawn in the heatmap. The statistical significance was analyzed by one-way ANOVA (*p* < 0.05) calculated with SPSS 21.0 software.

## 5. Conclusions

In summary, this study compared the rhizosphere microflora of different grape cultivars in northern China. The results showed that the richness and diversity of the bacterial and fungal communities in the root compartments were significantly influenced by the grape varieties. Different microbiota characteristics were found in the rhizosphere of different grape varieties; for instance, *Pseudomonas* and *Rhizobium*, considered as potential plant-growth-promoting bacteria, were more enriched in Pinot noir, and the potential ammonia-oxidizing *Nitrosospira* was enriched in Gem. Regarding fungi, *Fusarium* was more enriched in Longan, and *Sporormiella* was more enriched in Merlot, while *Gibberella* and *Pseudallescheria* were more enriched in Gem, and *Mortierella* was more abundant in Cabernet Sauvignon. However, the 16S rDNA functional prediction indicated that no significance differentiates among the varieties. The results provide valuable information for guiding the isolation and culture of microorganisms and have the potential to harness the power of the microbiome to improve plant production and health.

## Figures and Tables

**Table 1:** The alpha-diversity of bacterial communities.

	Coverage	ACE (10^3^)	Chao1 (10^3^)	Simpson (10^−2^)	Shannon
Zin	0.958 ± 0.018 ^a^	2.12 ± 0.167 ^a^^b^	2.02 ± 0.264 ^ab^	0.593 ± 0. 04 ^a^	6.09 ± 0.098 ^a^
Cab	0.960 ± 0.016 ^a^	2.16 ± 0.158 ^a^^b^	2.05 ± 0.131 ^ab^	0. 617 ± 0.07 ^a^	6.07 ± 0.069 ^a^
Cha	0.967 ± 0.007 ^a^	2.03 ± 0.165 ^ab^	2.04 ± 0.164 ^ab^	0.646 ± 0.05 ^a^	6.05 ± 0.071 ^a^
Syr	0.972 ± 0.011 ^a^	2.06 ± 0.163 ^ab^	2.07 ± 0.151 ^ab^	0.693 ± 0.05 ^a^	5.99 ± 0.083 ^a^
Mer	0.959 ± 0.005 ^a^	1.89 ± 0.110 ^a^	1.87 ± 0.128 ^a^	0.678 ± 0.06 ^a^	6.02 ± 0.080 ^a^
Gem	0.956 ± 0.013 ^a^	2.02 ± 0.154 ^ab^	1.89 ± 0.162 ^a^	0.658 ± 0.16 ^a^	6.05 ± 0.123 ^a^
Rie	0.966 ± 0.007 ^a^	2.07 ± 0.137 ^ab^	2.04 ± 0.146 ^ab^	0.677 ± 0.11 ^a^	5.99 ± 0.119 ^a^
Pin	0.978 ± 0.005 ^a^	2.25 ± 0.181 ^b^	2.29 ± 0.185 ^b^	0.789 ± 0.47 ^a^	5.99 ± 0.234 ^a^

Superscript different letters indicate a significant difference between different grape varieties.

**Table 2:** The alpha-diversity of fungal communities.

	Coverage	ACE (10^2^)	Chao1 (10^2^)	Simpson (10^−2^)	Shannon
Zin	0.997 ± 0.001 ^a^	9.63 ± 0.506 ^b^	9.76 ± 0.771 ^b^	3.64 ± 0.907 ^a^	4.14 ± 0.119 ^c^
Cab	0.999 ± 0.001 ^a^	7.28 ± 0.797 ^a^	7.54 ± 0.638 ^ab^	7.78 ± 2.76 ^ab^	3.57 ± 0.301 ^ab^
Cha	0.997 ± 0.001 ^a^	7.17 ± 0.834 ^a^	7.09 ± 0.848 ^a^	4.68 ± 0.956 ^ab^	4.02 ± 0.128 ^bc^
Syr	0.998 ± 0.001 ^a^	8.56 ± 0.305 ^ab^	8.77 ± 0.611 ^ab^	6.21 ± 2.79 ^ab^	3.87 ± 0.265a ^bc^
Mer	0.998 ± 0.001 ^a^	8.23 ± 0.837 ^ab^	8.23 ± 0.957 ^ab^	9.80 ± 4.51 ^b^	3.49 ± 0.339 ^a^
Gem	0.998 ± 0.001 ^a^	7.30 ± 0.628 ^a^	7.40 ± 0.669 ^a^	4.84 ± 1.96 ^ab^	3.88 ± 0.250 ^abc^
Rie	0.998 ± 0.001 ^a^	7.66 ± 0.989 ^a^	7.83 ± 0.975 ^ab^	5.64 ± 0.963 ^ab^	3.80 ± 0.126 ^abc^
Pin	0.996 ± 0.003 ^a^	8.33 ± 1.47 ^ab^	8.12 ± 1.63 ^ab^	8.97 ± 2.95 ^ab^	3.57 ± 0.181 ^ab^
Lon	0.998 ± 0.001 ^a^	9.13 ± 0.503 ^ab^	9.06 ± 0.525 ^ab^	4.74 ± 0.313 ^ab^	3.97 ± 0.117 ^abc^

Superscript different letters indicate a significant difference between different grape varieties.

**Table 3:** The taxonomic metric (calculated from OTU) for bacterial diversity patterns.

		Bray–Curtis				Unweighted Unifrac			
		ADONIS		ANOSIM		ADONIS		ANOSIM	
Group	Factor	R^2^	*p*	R	*p*	R^2^	*p*	R	*p*
Eight	Variety	0.319	0.001	0.317	0.001	0.215	0.001	0.245	0.001

**Table 4:** The taxonomic metric (calculated from OTU) for fungal diversity patterns.

		Bray–Curtis				Unweighted Unifrac			
		ADONIS		ANOSIM		ADONIS		ANOSIM	
Group	Factor	R^2^	*p*	R	*p*	R^2^	*p*	R	*p*
Nine	Variety	0.373	0.001	0.295	0.001	0.442	0.001	0.401	0.001

## Data Availability

The sequencing data sets of bacteria and fungi were deposited in the NCBI’s Sequence Read Archive with the Accession Numbers SRP216299 and SRP216297, respectively.

## Supplementary Materials

The following supporting information can be downloaded at: https://www.mdpi.com/article/10.3390/plants11091111/s1, Figure S1: The rarefaction curves of bacteria (a) and fungi (b); Figure S2: KEGG orthologs (KOs) identified the key genotypic features. The 32 most prevalent ECs, standardized by Z-score across all data sets, and the stars represents the relatives of enzyme larger than 3.0%; Table S1: Number of sequences, total OTUs in bacteria and fungi; Table S2: Indices of co-networks; Table S3: Bacterial sequences aligned using BLAST from the NCBI nucleotide database; Table S4: Fungal sequences aligned using BLAST from the NCBI nucleotide database; Table S5: Analysis of soil physical and chemical indexes of the rhizosphere soils; Table S6: Physio-chemical characteristics of the soil samples; Table S7: Air condition in the sampling site; Table S8: KEGG orthologs (KOs) identified the key genotypic features. Relative abundance pathways in KEGG level 2 in the grapevine rhizosphere; Table S9: KEGG orthologs (KOs) identified the key genotypic features. Relative abundance pathways in KEGG level 3 in the grapevine rhizosphere.
