# Supplementary material for: Grape Cultivar Features Differentiate the Grape Rhizosphere Microbiota
Source: Plants (Basel). 2022 Apr 20;11(9):1111. doi: 10.3390/plants11091111 (PMC9102929; doi:10.3390/plants11091111)
Supplement: Supplementary file 1 [file plants-11-01111-s001.zip › plants-1665618-supplementary.pdf]

Table S1. Number of sequences, total OTUs in bacteria and fungi

		Lon (10^3)	Zin (10^3)	Cab (10^3)	Cha (10^3)	Syr (10^3)	Mer (10^3)	Gem (10^3)	Rie (10^3)	Pin (10^3)
**Bacteria**	**Sequence**	--	52.27±20.87	52.98±20.45	59.34±15.85	77.50±38.31	47.19±9.34	44.10±21.70	47.50±8.75	64.99±11.06
	**OTUs**	--	3.89±0.95	3.97±0.74	4.19±0.59	4.45±0.71	3.72±0.36	3.48±0.82	3.84±0.39	4.49±0.43
**Fungi**	**Sequence**	75.62±27.91	97.78±118.78	53.56±26.06	96.38±25.58	100.07±25.74	71.88±27.44	77.82±31.79	68.73±48.55	79.70±24.48
	**OTUs**	0.89±0.22	1.09±0.20	1.00±0.26	1.31±0.13	1.29±0.12	1.08±0.27	1.24±0.25	1.07±0.37	1.17±0.18

Table S2. Indices of co-networks

	Nodes	Edges	Disgree distribution	Betweenness Centrality Distribution	Density	Modularity
**Bacteria**	1384	6973	10.077	4	0.007	0.073
**Fungi**	1020	4038	7.918	4	0.008	0.151

Table S3. Bacterial sequences aligned using BLAST from the NCBI nucleotide database.

OTU No.	References species	Accession.No	Similarity
**OTU130**	*Arthrobacter* sp.	MG860374.1	100
**OTU375**	Uncultured bacterium	MF113989.1	100
**OTU646**	*Bacillus* sp.	MK414968.1	100
**OTU795**	*Steroidobacter* sp.	JF515710.1	100
**OTU1182**	Uncultured bacterium	LC026882.1	100
**OTU1203**	*Kocuria*	MK696247.1	100
**OTU1226**	*Bradyrhizobium* sp.	FJ380996.1	100
**OTU1496**	*Romboutsia* sp.	LT996174.1	100
**OTU2150**	Uncultured bacterium	KM328768.1	100
**OTU2257**	*Blastococcus* sp.	MK696404.1	100
**OTU2425**	*Arthrobacter* sp.	MG860431.1	100
**OTU2536**	*Arthrobacter* sp.	MK696393.1	100
**OTU3419**	*Arthrobacter* sp.	KX171688.1	100
**OTU3429**	Uncultured bacterium	MH445072.1	100
**OTU3574**	*Arthrobacter* sp.	MK053924.1	100

**OTU3775**	Uncultured Actinobacterium	KJ081636.1	100
**OTU3794**	Nocardioides（family）	MK138625.1	100
**OTU3854**	Planomicrobium	MH550146.1	100

Table S4. Fungal sequences aligned using BLAST from the NCBI nucleotide database.

OTU No.	References species	Accession. No	Similarity(%)
**OTU104**	*Cladosporium* sp.	MG569541.1	100
**OTU232**	Nectriaceae	HG936943.1	100
**OTU252**	Uncultured fungi	KU931455.1	100
**OTU445**	Tremellomycetes	HG935384.1	100
**OTU765**	*Alternaria* sp.	MK461092.1	100
**OTU858**	Solicoccozyma	KY105435.1	100
**OTU891**	Uncultured fungi	MF148734.1	100
**OTU896**	*Alternaria* sp.	MK461092.1	100
**OTU1019**	*Alternaria* sp.	MK461092.1	100
**OTU1146**	Phoma	KT150684.1	99.61
**OTU1289**	Ascochyta	KF293988.1	100
**OTU1514**	Thyrostroma sp	MG020338.1	95.31
**OTU1601**	Uncultured fungi	JX390488.1	100
**OTU1683**	*Alternaria* sp.	MK461092.1	100
**OTU1730**	*Fusarium* sp.	MF509747.1	100

**OTU1732**	Tremellomycetes	HG935384.1	100
**OTU1736**	*Mortierella* sp.	MK281958.1	98.38
**OTU1737**	*Chaetomium* sp.	KY132110.1	100
**OTU1740**	Uncultured fungi	KU931358.1	100
**OTU1781**	Uncultured fungi	KU931455.1	100
**OTU1795**	Uncultured fungi	JX333053.1	100
**OTU1800**	Uncultured fungi	JX359333.1	100
**OTU1819**	*Fusarium* sp.	KJ690090.1	100
**OTU1859**	*Alternaria* sp.	MK461092.1	100
**OTU1941**	*Alternaria* sp.	MK461092.1	100
**OTU1975**	Uncultured fungi	KU534664.1	100
**OTU1999**	Uncultured fungi	GQ225148.1	100
**OTU2024**	Uncultured fungi	JX984789.1	98
**OTU2025**	Chaetomium	KY132110.1	100
**OTU2094**	Neonectria	MF782762.1	100
**OTU2104**	Uncultured fungi	JX333053.1	100
**OTU2180**	Tremellomycetes	HG935384.1	100

**OTU2249**	Tremellomycetes	HG935384.1	100
**OTU2346**	Uncultured fungi	JX352646.1	100
**OTU2352**	Uncultured fungi	GQ225160.1	100
**OTU2365**	Acremonium	KM816680.1	100

Table S5. Analysis of soil physical and chemical indexes of the rhizosphere soils

Name	pH	Salty	conductivity（μm-1· cm）
**Cab**	7.58	70.97	400.0
**Mer**	7.38	92.71	566.0
**Pin**	7.78	90.71	285.0
**Gem**	7.64	47.81	287.0
**Zin**	7.78	47.96	398.0
**Syr**	7.70	47.18	210.0
**Lon**	8.37	48.34	131.8
**Rie**	8.20	47.26	135.9
**Cha**	7.78	71.48	220.0

Table S6. Physico-chemical characteristics of the soil samples.

Item	Content (%)	Detection method
**Organic matter**	67.6	NY/T 1121.6-2006
**Total nitrogen**	2.35	NY/T 53-1987
**Available phosphorus**	1.26	GB/T 17141-1997
**Available potassium**	1.40	NY/T 889-2004

Table S7. Air condition in the sampling site

Tem (℃)	AQI	$PM_{2.5}$ (μg. m^{-3})	PM_{10} (μg. m^{-3})	SO_2 (μg. m^{-3})	CO (mg. m^{-3})	NO_2 (μg. m^{-3})	O_3_8 h (μg. m^{-3})
16-29	63	12	40	10	0.4	13	116

Table S8. KEGG orthologs (KOs) identified the key genotypic features. Relative abundance pathways in KEGG level 2 in the grapevine rhizosphere.

Metabolism	Zin	Gem	Rie	Syr	Mer	Cha	Cab	Pin
Membrane Transport	12.21%	12.07%	12.48%	11.96%	12.08%	12.34%	12.29%	12.37%
Amino Acid Metabolism	11.41%	11.37%	11.32%	11.38%	11.46%	11.41%	11.47%	11.28%
Carbohydrate Metabolism	10.83%	10.84%	10.85%	10.80%	10.91%	10.88%	10.94%	10.74%
Replication and Repair	6.79%	6.76%	6.79%	6.85%	6.79%	6.75%	6.76%	6.80%
Energy Metabolism	5.48%	5.52%	5.41%	5.51%	5.50%	5.48%	5.45%	5.44%
Poorly Characterized	4.87%	4.91%	4.91%	4.91%	4.87%	4.85%	4.85%	4.93%
Xenobiotics Biodegradation and Metabolism	4.56%	4.52%	4.49%	4.47%	4.59%	4.58%	4.60%	4.43%
Lipid Metabolism	4.18%	4.20%	4.11%	4.13%	4.21%	4.17%	4.19%	4.10%
Metabolism of Cofactors and Vitamins	4.14%	4.14%	4.08%	4.17%	4.14%	4.12%	4.12%	4.11%
Translation	3.97%	3.96%	3.92%	4.02%	3.96%	3.94%	3.94%	3.96%
Nucleotide Metabolism	3.14%	3.13%	3.14%	3.17%	3.15%	3.13%	3.14%	3.14%
Cellular Processes and Signaling	3.11%	3.11%	3.15%	3.17%	3.07%	3.07%	3.05%	3.19%
Cell Motility	2.55%	2.61%	2.60%	2.64%	2.54%	2.56%	2.51%	2.66%

Metabolism	2.47%	2.47%	2.49%	2.51%	2.47%	2.48%	2.47%	2.48%
Metabolism of Terpenoids and Polyketides	2.48%	2.49%	2.44%	2.45%	2.50%	2.48%	2.49%	2.44%
Transcription	2.37%	2.38%	2.38%	2.37%	2.37%	2.37%	2.37%	2.37%
Folding, Sorting and Degradation	2.14%	2.15%	2.13%	2.16%	2.14%	2.13%	2.13%	2.14%
Genetic Information Processing	2.07%	2.07%	2.12%	2.12%	2.06%	2.06%	2.04%	2.15%
Metabolism of Other Amino Acids	2.07%	2.06%	2.05%	2.05%	2.06%	2.07%	2.07%	2.05%
Signal Transduction	2.00%	2.03%	2.03%	1.98%	1.98%	2.00%	1.98%	2.04%
Enzyme Families	1.85%	1.86%	1.85%	1.86%	1.85%	1.84%	1.85%	1.84%
Glycan Biosynthesis and Metabolism	1.49%	1.53%	1.48%	1.51%	1.47%	1.46%	1.46%	1.52%
Biosynthesis of Other Secondary Metabolites	1.04%	1.04%	1.02%	1.03%	1.04%	1.04%	1.04%	1.01%
others	2.81%	2.79%	2.79%	2.80%	2.79%	2.80%	2.80%	2.81%

Table S9. KEGG orthologs (KOs) identified the key genotypic features. Relative abundance pathways in KEGG level 3 in the grapevine rhizosphere.

Metabolism	Zin	Gem	Rie	Syr	Mer	Cha	Cab	Pin
Transporters	6.26%	6.15%	6.38%	6.06%	6.19%	6.34%	6.32%	6.28%
ABC transporters	3.84%	3.80%	3.90%	3.72%	3.79%	3.90%	3.87%	3.86%
General function prediction only	3.42%	3.46%	3.43%	3.44%	3.43%	3.41%	3.41%	3.43%
DNA repair and recombination proteins	2.31%	2.30%	2.32%	2.33%	2.32%	2.30%	2.31%	2.31%
Two-component system	1.87%	1.91%	1.91%	1.86%	1.86%	1.88%	1.86%	1.92%
Purine metabolism	1.89%	1.88%	1.88%	1.89%	1.89%	1.88%	1.88%	1.89%
Ribosome	1.61%	1.61%	1.59%	1.63%	1.61%	1.60%	1.60%	1.60%
Function unknown	1.45%	1.45%	1.49%	1.47%	1.44%	1.44%	1.44%	1.50%
Peptidases	1.43%	1.43%	1.42%	1.45%	1.43%	1.42%	1.43%	1.42%
Transcription factors	1.40%	1.39%	1.44%	1.39%	1.39%	1.40%	1.40%	1.43%
Secretion system	1.38%	1.40%	1.42%	1.43%	1.38%	1.38%	1.37%	1.44%
Bacterial motility proteins	1.31%	1.35%	1.33%	1.36%	1.31%	1.31%	1.29%	1.36%
Butanoate metabolism	1.33%	1.34%	1.32%	1.31%	1.35%	1.34%	1.35%	1.30%

Oxidative phosphorylation	1.31%	1.32%	1.27%	1.32%	1.31%	1.30%	1.29%	1.29%
Arginine and proline metabolism	1.27%	1.27%	1.27%	1.27%	1.27%	1.28%	1.28%	1.27%
Pyrimidine metabolism	1.26%	1.26%	1.25%	1.27%	1.26%	1.25%	1.26%	1.26%
Valine, leucine and isoleucine degradation	1.21%	1.22%	1.20%	1.19%	1.23%	1.22%	1.23%	1.19%
Propanoate metabolism	1.19%	1.20%	1.18%	1.17%	1.21%	1.20%	1.21%	1.17%
Pyruvate metabolism	1.17%	1.18%	1.18%	1.18%	1.18%	1.18%	1.18%	1.17%
Amino acid related enzymes	1.16%	1.16%	1.15%	1.17%	1.16%	1.16%	1.15%	1.16%
Glycolysis / Gluconeogenesis	1.12%	1.12%	1.13%	1.12%	1.13%	1.13%	1.13%	1.11%
Carbon fixation pathways in prokaryotes	1.09%	1.10%	1.07%	1.10%	1.10%	1.09%	1.09%	1.08%
Methane metabolism	1.08%	1.09%	1.07%	1.08%	1.10%	1.09%	1.08%	1.06%
Chromosome	1.08%	1.08%	1.08%	1.10%	1.07%	1.07%	1.06%	1.09%
Fatty acid metabolism	1.03%	1.04%	1.02%	1.01%	1.05%	1.04%	1.05%	1.01%
Ribosome Biogenesis	0.99%	0.99%	0.99%	1.00%	0.99%	0.98%	0.98%	1.00%
Others	0.96%	0.96%	0.97%	1.01%	0.97%	0.97%	0.97%	0.98%
Glycine, serine and threonine metabolism	0.97%	0.96%	0.97%	0.97%	0.98%	0.97%	0.97%	0.97%
Amino sugar and nucleotide sugar metabolism	0.96%	0.96%	0.96%	0.96%	0.96%	0.96%	0.96%	0.95%
Other ion-coupled transporters	0.95%	0.93%	0.99%	0.97%	0.95%	0.94%	0.94%	0.98%

Alanine, aspartate and glutamate metabolism	0.94%	0.93%	0.93%	0.93%	0.94%	0.94%	0.94%	0.93%
Aminoacyl-tRNA biosynthesis	0.91%	0.90%	0.89%	0.91%	0.90%	0.90%	0.90%	0.89%
Citrate cycle (TCA cycle)	0.89%	0.90%	0.88%	0.89%	0.90%	0.89%	0.90%	0.88%
Lipid biosynthesis proteins	0.89%	0.89%	0.86%	0.88%	0.89%	0.88%	0.88%	0.87%
Porphyrin and chlorophyll metabolism	0.87%	0.88%	0.85%	0.87%	0.87%	0.88%	0.87%	0.87%
Tryptophan metabolism	0.85%	0.85%	0.84%	0.83%	0.86%	0.86%	0.86%	0.83%
Glyoxylate and dicarboxylate metabolism	0.84%	0.84%	0.84%	0.82%	0.84%	0.84%	0.84%	0.84%
Transcription machinery	0.84%	0.86%	0.81%	0.84%	0.85%	0.84%	0.84%	0.82%
Energy metabolism	0.81%	0.81%	0.81%	0.80%	0.80%	0.81%	0.80%	0.81%
Benzoate degradation	0.79%	0.79%	0.79%	0.77%	0.81%	0.80%	0.81%	0.77%
Chaperones and folding catalysts	0.77%	0.78%	0.77%	0.78%	0.76%	0.76%	0.76%	0.78%
Cysteine and methionine metabolism	0.77%	0.76%	0.76%	0.77%	0.76%	0.76%	0.76%	0.76%
Valine, leucine and isoleucine biosynthesis	0.75%	0.76%	0.74%	0.76%	0.76%	0.75%	0.76%	0.75%
DNA replication proteins	0.75%	0.75%	0.76%	0.76%	0.75%	0.75%	0.75%	0.76%
Nitrogen metabolism	0.75%	0.75%	0.75%	0.74%	0.75%	0.75%	0.74%	0.75%
Protein folding and associated processing	0.70%	0.70%	0.70%	0.70%	0.69%	0.70%	0.69%	0.70%
Lysine degradation	0.69%	0.69%	0.68%	0.67%	0.69%	0.69%	0.69%	0.67%

Pentose phosphate pathway	0.66%	0.66%	0.67%	0.66%	0.66%	0.66%	0.66%	0.67%
Phenylalanine, tyrosine and tryptophan biosynthesis	0.64%	0.64%	0.63%	0.65%	0.64%	0.64%	0.64%	0.64%
beta-Alanine metabolism	0.64%	0.64%	0.63%	0.62%	0.65%	0.64%	0.65%	0.63%
Homologous recombination	0.64%	0.63%	0.63%	0.65%	0.64%	0.63%	0.63%	0.64%
Aminobenzoate degradation	0.64%	0.64%	0.62%	0.62%	0.64%	0.64%	0.64%	0.61%
Bacterial secretion system	0.62%	0.62%	0.65%	0.64%	0.61%	0.62%	0.62%	0.66%
others	34.08%	34.08%	33.94%	34.19%	34.04%	33.98%	33.99%	34.04%

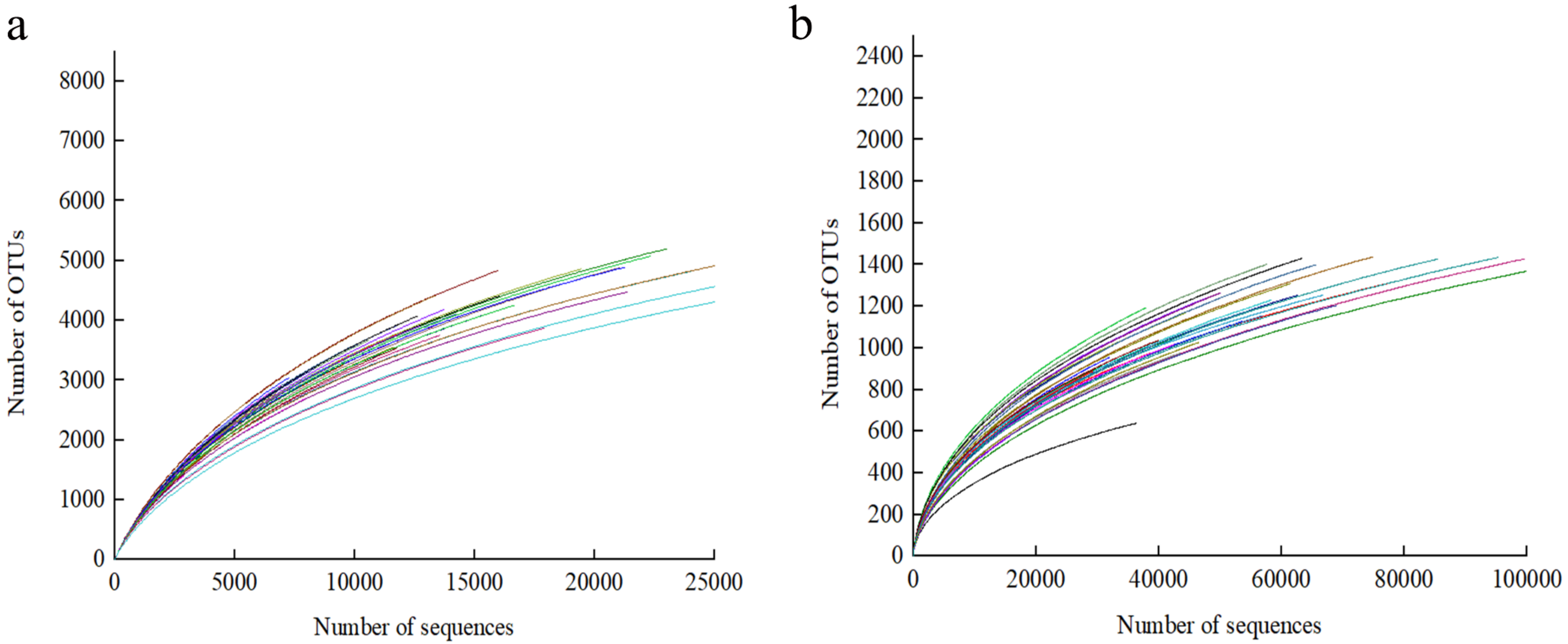

Figure S1. The rarefaction curves of bacteria (a) and fungi (b).

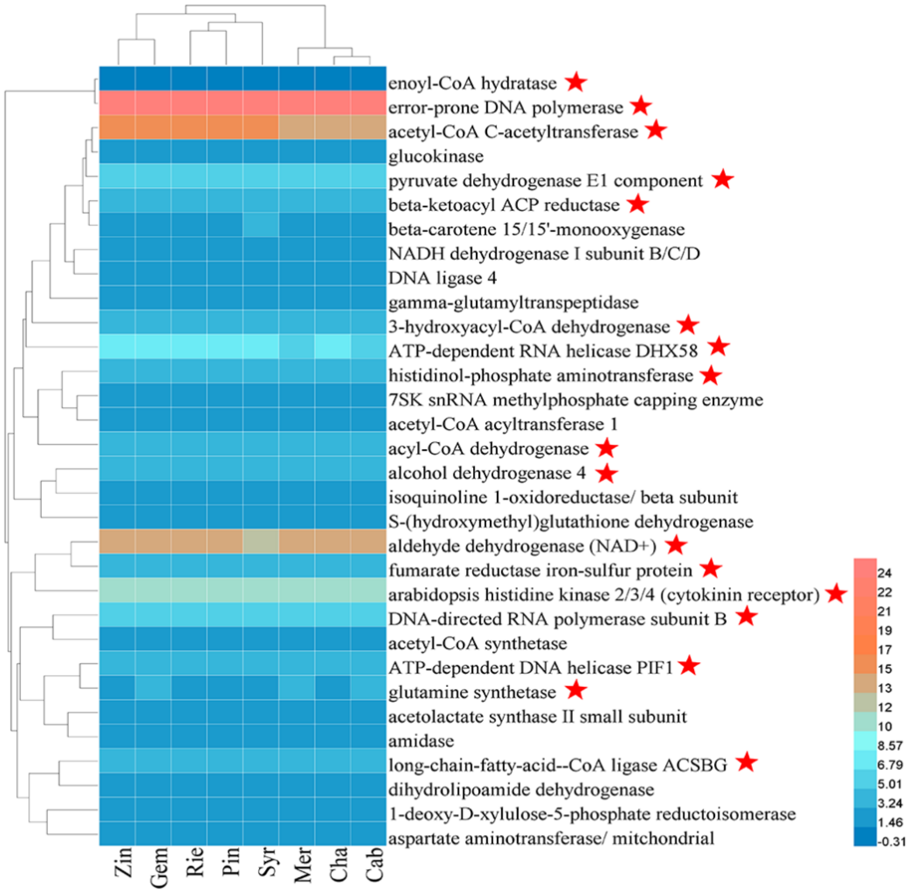

Figure S2. KEGG orthologs (KOs) identified the key genotypic features. The 32 most prevalent ECs, standardized by Z-score across all data sets, and the stars represents the relatives of enzyme larger than 3.0%.
